# Surgery

**Published:** 1905-09-23

**Authors:** 


### SURGERY.

**Thoracic Surgery.**—There can be no doubt that the thorax now offers greater difficulties to the surgeon in the treatment of disease than any other region of the body. But there are signs that some of these difficulties may yet be overcome. It was in 1879 that Estlander brought forward his operation for the radical cure of chronic empyema. And some time later Schede proposed not only to resect portions of the ribs, but also to remove all the other constituents of the chest wall except the skin and superficial muscles which lie over the cavity to be closed. This operation has not been adopted much because of its formidable character, but Ballance[1] reports seven of his own cases with two deaths. One of the fatal cases was a pyo-pneumothorax and the other died from a cerebral abscess. The parietal pleura in these chronic cases forms a hard mass often half-an-inch thick, and it is the removal of this which holds out a better chance of the obliteration of the empyema cavity than can be obtained by Estlander's method. Delorme's operation attempts to deal with the visceral pleura, merely turning up a large flap containing the whole thickness of the chest wall. The thick pleura covering the lung is then stripped off (decortication of lung) and the lung can then expand to its former dimensions. The liability to injure the lung and the chances of infection of the fresh surface of the lung which is exposed have deterred surgeons from attempting this operation with any frequency. But apart from conditions, such as empyema, which shut off a part of the pleural cavity from the rest of the chest, very little has been possible surgically hitherto because of the difference between the intra- and extra-thoracic air pressure.[2] But now in the clinic of v. Mikulicz experiments have been undertaken with a view to overcoming this difficulty. In order to prevent the collapse of the lung, which inevitably occurs when the chest wall is opened under ordinary conditions, the operation is conducted in a full-sized pneumatic cabinet. The patient's body is within the cabinet but the head projects outside through an opening in the wall made to fit closely to the neck by means of a perforated curtain of rubber. The lower part of the patient's body, too, below the waist is enclosed in a rubber bag communicating with the outer air. The cabinet is large enough to contain the operator and his assistants and apparatus. The atmospheric pressure within the cabinet is a negative one, and is 10 milligrammes of mercury less than the atmospheric pressure; this is sufficient to prevent any collapse of the lung when the pleural cavity is freely opened. No inconvenience is experienced by those within the cabinet, for the decrease in pressure is no greater than that regularly existing at a height of 300 metres. Ten operations, the details of which are not given, have already been successfully performed by this method.

**Modern Methods of Diagnosis and Treatment of Fractures.**—There is probably no class of surgical cases in which accuracy in diagnosis and treatment is more important than in that of fractures. And in both these respects great advance has been made recently, both by the application of the principles of aseptic surgery and by the use of the Röntgen rays. There can be no doubt that the ideal method of diagnosis is that every case should be submitted to the $x$-rays, whether uncertainty as to diagnosis exists or not. In many apparently simple cases an unsuspected obliquity or comminution in the fracture is seen which greatly modifies the treatment. Gilchrist[3] mentions several of such cases. In one, the ankle was the seat of a severe smash with much swelling and displacement. It was regarded as a comminuted fracture and the shortening was left unreduced. But the fluorescent screen showed that it was merely an oblique fracture of both bones and the fracture was actually reduced under the $x$-rays. And many other examples are given by the same author in which complicated fractures have been set under the actual guidance of the rays. But in certain situations only a small part of the difficulty of the case has been surmounted when the diagnosis has been made clear. For instance, in fractures of the patella and of the neck of the femur, however accurately the displacement is recognised, the treatment by any of the old methods is very unsatisfactory. In the case of the patella, it has long been recognised that only the open method of operating gives any chance of good results in the majority of cases in which there is a distinct separation of the fragments. Hutchinson[4] reviews the whole question, giving the opinions of many modern authorities, and describing seven cases of his own. It is quite clear that the main obstacle to bony union in these cases is not merely wide devarication, tilting of the fragments, or interposition of blood-clot, though all these factors doubtless play a part. It is the falling down over the fractured surfaces of the torn aponeurosis. And however accurately the fragments may be brought together, unless this intervening fibrous membrane be removed, bony union is very unlikely to occur. So that now the routine method of treatment of fracture of the patella with distinct separation of the fragments should be by an open operation, making a semi-lunar incision through the skin with the convexity downwards. The blood-clot is washed out with saline solution and the fragments wired together with silver or brass wire, after cutting off the ragged edge of aponeurosis. Authorities differ as to the best time for the performance of this operation. The majority wait for a week after the accident, until the swelling and inflammation have subsided. Others, however, prefer to act as soon as the nature of the injury is recognised. In fractures of the neck of the femur another instance of inadequate methods of treatment presents itself, but in this case, the advantages of the new devices over the old are not nearly so clear. Freeman,[5] before describing a case in which he employed an open operation, dwells on the difficulty of procuring bony union and of avoiding shortening. As long ago as 1858 an attempt was made to unite the fractured neck of the femur by an open operation, the patient dying of hospital gangrene. The first successful case occurred in 1888, but there have been only 14 published since. Parkhill[6] introduced an apparatus consisting of three screws which are joined together by a metal frame, and which are screwed into the neck and shaft of the bone. Freeman also uses three screws, but depends on a wooden clamp to fix them in relation to one another. The great difficulty encountered is the porous and friable nature of the neck of the femur. But even in America where the method is most practised, surgeons are by no means unanimous in their approval of it. Ochsner and Ruth[7] contend that if the limb is put up, not only with longitudinal extension but also in a position of abduction and internal rotation, that as good results follow as attend the treatment of fractures in the shafts of long bones. There is one small fracture which is probably of common occurrence but the existence of which could seldom be proved before the advent of skiagraphy. This is fracture of the tibial tuberosity. Ware[8] describes and figures a typical case of this injury and reports of such cases are becoming increasingly common in surgical literature. It is often sufficient to immobilise the knee and bandage firmly for several weeks, but it is better sometimes to expedite recovery by an open operation, removing the fragment of bone or pegging it down to the tibia.

[1] Brit. Med. Jour., Dec. 10. [2] Med. Rec., Nov. 5. [3] New York Med. Jour., Oct. 22. [4] Annals of Surg., Oct. [5] Ibid. [6] Ibid. [7] Ibid. [8] bid. Nov.

